# A Mixture of Formic Acid, Benzoic Acid, and Essential Oils Enhanced Growth Performance via Modulating Nutrient Uptake, Mitochondrion Metabolism, and Immunomodulation in Weaned Piglets

**DOI:** 10.3390/antiox13020246

**Published:** 2024-02-19

**Authors:** Xinyu Wang, Tanyi Deng, Xuemei Zhou, Licui Chu, Xiangfang Zeng, Shihai Zhang, Wutai Guan, Fang Chen

**Affiliations:** 1 College of Animal Science and National Engineering Research Center for Pig Breeding Industry, South China Agricultural University, Guangzhou 510642, China; dengtanyi@stu.scau.edu.cn (T.D.); 20010114@stu.scau.edu.cn (X.Z.); 2543872938@stu.scau.edu.cn (L.C.); zhangshihai@scau.edu.cn (S.Z.); wtguan@scau.edu.cn (W.G.); 2 Guangdong Laboratory of Modern Agriculture in Lingnan, Guangzhou 510642, China; 3 State Key Laboratory of Animal Nutrition, Ministry of Agriculture Feed Industry Centre, China Agricultural University, Beijing 100193, China; b20213040352@cau.edu.cn (X.W.); zengxf@cau.edu.cn (X.Z.)

**Keywords:** formic acid, benzoic acid, essential oil, intestinal barrier, antioxidant, gut microbiota

## Abstract

This study aimed to evaluate the effects of a complex comprising formic acid, benzoic acid, and essential oils (AO3) on the growth performance of weaned piglets and explore the underlying mechanism. Dietary AO3 supplementation significantly enhanced the average daily gain (ADG) and average daily feed intake (ADFI), while decreasing the feed conversion rate (FCR) and diarrhea rate (*p* < 0.05). Additionally, AO3 addition altered the fecal microflora composition with increased abundance of f_Prevotellaceae. LPS challenges were further conducted to investigate the detailed mechanism underlying the benefits of AO3 supplementation. The piglets fed with AO3 exhibited a significant increase in villus height and decrease in crypt depth within the jejunum, along with upregulation of ZO-1, occludin, and claudin-1 (*p* < 0.05) compared with those piglets subjected to LPS. Furthermore, AO3 supplementation significantly ameliorated redox disturbances (T-AOC, SOD, and GSH) and inflammation (TNF-α, IL-1β, IL-6, and IL-12) in both the serum and jejunum of piglets induced by LPS, accompanied by suppressed activation of the MAPK signaling pathway (ERK, JNK, P38) and NF-κB. The LPS challenge downregulated the activation of the AMPK signaling pathway, mRNA levels of electron transport chain complexes, and key enzymes involved in ATP synthesis, which were significantly restored by the AO3 supplementation. Additionally, AO3 supplementation restored the reduced transport of amino acids, glucose, and fatty acids induced by LPS back to the levels observed in the control group. In conclusion, dietary AO3 supplementation positively affected growth performance and gut microbiota composition, also enhancing intestinal barrier integrity, nutrient uptake, and energy metabolism, as well as alleviating oxidative stress and inflammation under LPS stimulation.

## 1. Introduction

Weaning piglets undergo a challenging transition characterized by diarrhea, growth inhibition, and increased mortality, leading to significant economic losses [1,2]. The root cause of these adverse effects can be primarily ascribed to gastrointestinal disorders triggered by alterations in physiological, immune, and microbial factors during this process [3]. To counteract the detrimental impacts of weaning stress, antibiotic growth promoters (AGPs) have been extensively adopted to mitigate post-weaning diarrhea (PWD) and enhance growth. Nevertheless, the prohibition of growth-promoting antibiotics as feed additives has been implemented in many countries, necessitating the identification and development of effective alternatives to AGPs [4,5].

Organic acids, representing a class of natural, non-toxic acidic organic compounds, have been reported as a promising substitute for AGPs [6,7]. Numerous pieces of evidence have shown that dietary supplementation with individual or combined organic acids, such as citric acid, benzoic acid, and fumaric acid, could effectively alleviate weaning stress and improve the growth performance of piglets [8]. Other promising alternatives to AGP are essential oils, typically extracted from various plants [9,10]. Key constituents in essential oils, such as thymol and cinnamaldehyde, have demonstrated significant potential in enhancing animal health by improving gut integrity, reducing inflammation, and providing protection against harmful pathogens [11].

While both plant-derived organic acids and essential oils can function as substitutes in weaning piglet diets, their underlying mechanisms diverge. Organic acids operate by reducing intestinal pH, thereby inhibiting the growth of harmful bacteria, enhancing the digestion of dietary nutrients, and improving intestinal enzyme activity [12]. The antibacterial mechanism of essential oils involves damaging the bacterial cell membrane or penetrating the bacterial interior to disrupt its physiological processes [13,14]. Some studies suggest a synergistic effect may enhance the effectiveness when organic acids and plant essential oils are used in combination in various species, including piglets, broilers, and rabbits [15,16,17,18]. However, a significant proportion of these studies have primarily focused on the combined effects of benzoic acid and essential oils, with limited reporting on the outcomes of diverse combinations involving various acids and essential oils. This oversight may lead to the potential synergies among different acids being overlooked, especially neglecting the pronounced antimicrobial effects resulting from the high acidity of formic acid [19]. Therefore, we hypothesized that a combination of formic acid, benzoic acid, and plant essential oils could significantly influence the intestinal function and overall health of weaned piglets, thereby positively affecting their growth performance.

In this study, we initially evaluated the effects of a blend containing formic acid, benzoic acid, and plant essential oils on the growth performance of weaned piglets. Subsequently, we delved into the underlying mechanisms of these observed benefits by inducing stress using an LPS challenge to mimic the acute stress response triggered by weaning stress. We systematically examined the protective effects on various parameters, including intestinal morphology, redox homeostasis, inflammation, barrier function, nutrient absorption, and energy metabolism. Our research results shed light on a promising approach to enhance intestinal health through the effective utilization of combined substitutes.

## 2. Materials and Methods

### 2.1. Animals and Experimental Design

**Figure 1:**
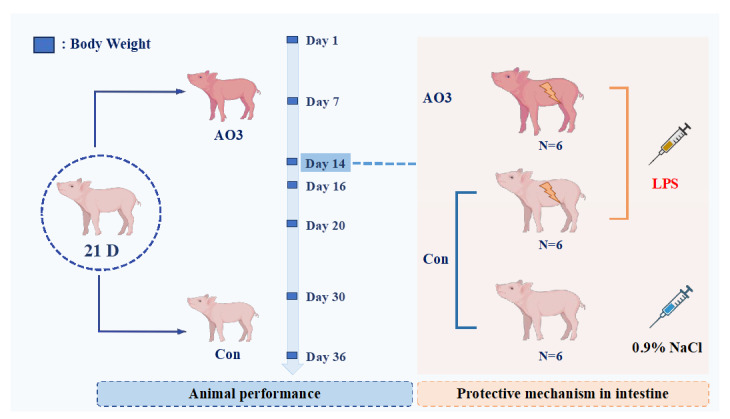
Arrangement of animal experiments. A total of 360 21-day-old Duroc × Yorkshire × landrace weaned piglets (6.59 ± 1.06 kg) were randomly divided into 2 groups with 9 replicates per group and 20 pigs per replicate. The trial lasted 36 days and was divided into two phases; the first phase lasted from days 1–14 and the second phase days 15–36. On the 14th day of the feeding experiment, 18 piglets were selected, including 12 control piglets and 6 treated piglets. Of these, 6 control piglets were injected with normal saline, and the remaining 6 control piglets and 6 treated piglets (AO3 group) were injected with LPS. Blood and tissue samples were collected 4 h after LPS injection.

The animal procedures employed in this experiment were approved by the South China Agricultural University Animal Care and Use Committee. The arrangement of animal experiments is shown in Figure 1. A total of 360 21-day-old Duroc × Yorkshire × landrace weaned piglets (6.59 ± 1.06 kg) were randomly divided into 2 groups with 9 replicates per group and 20 pigs per replicate. The trial lasted 36 days and was divided into two phases; the first phase lasted from days 1 to 14 and the second phase lasted from days 15 to 36. After the initial stage (days 1–14), a subset of piglets consisting of 18 individuals (12 in the control group and 6 in the AO3 group) were chosen for the LPS challenge test. The remaining piglets proceeded with the ongoing growth performance assessments. The diet formula and the composition of nutrition are presented in Table 1, and were compliant with the recommendations of the NRC (2012) [20]. The arrangement of the animal experiment is shown in Figure 1. In the first stage, the diets were as follows: A—basal diet (without organic acids and essential oils), B—basal diet + 2500 mg/ kg of AO3. In the second stage, the diets were as follows: A—basal diet (without organic acids and essential oils), B—basal diet +1800 mg/kg of AO3. The AO3 is a commercial product sourced from DSM (Shanghai, China). The composition of the AO3 product primarily includes formic acid (31.5%), benzoic acid (55.5%), and essential oils (13.7%).

### 2.2. LPS Challenge Model Establishment

After the first phase (day 1–14), a total of 18 piglets (12 in the control group and 6 in the AO3 group) were selected for the LPS challenge experiment. The piglets were divided into three distinct groups based on their dietary composition and injection treatment (Figure 1). The control group (CON) comprised 6 piglets receiving a basal diet and an intraperitoneal injection of saline. Including a saline injection as a control group in the LPS challenge experiment helped us identify differences induced by LPS rather than injection stimulation, thereby enhancing the reliability and validity of the study results. The LPS group (LPS) included six piglets on the basal diet, subjected to intraperitoneal injection with LPS (*Escherichia coli* 055: B5, Sigma-Aldrich St. Louis, MO, USA) at 100 μg/kg body weight (BW). The inclusion of the LPS group aimed to observe stress damage resulting from LPS challenge in piglets without additional essential oil organic acids. The LPS+AO3 group (LPS+AO3) consisted of 6 piglets receiving an AO3-supplemented diet along with an intraperitoneal injection of LPS at 100 μg/kg BW. Blood and tissue samples were collected 4 h after LPS injection. The purpose of this group was to assess the potential impact of AO3 supplementation on the immune response of piglets under conditions of LPS-induced immune stress.

### 2.3. Performance and Incidence of Diarrhea

On days 1, 7, 16, 20, 30, and 36 of the experiment, the litter weight of piglets was measured and the average daily gain (ADG) of weaned piglets was calculated. The feed intakes on days 1–7, 8–15, 16–20, 21–30, and 31–36 were recorded, the average daily feed intake (ADFI) of the weaned piglets was calculated, and the feed conversion rate (FCR) at each stage was calculated. Twice a day, a trained person evaluated the occurrence of diarrhea based on the classification of feces in the enclosure. ADG was determined by the formula (total weight at the end of the experiment − total weight at the beginning of the experiment)/(number of piglets × number of experimental days); ADFI was calculated as total feed intake/(number of piglets × number of experimental days). FCR was calculated as follows: FCR = average daily feed intake (ADFI)/average daily gain (ADG). Diarrhea frequency = number of diarrhea piglets/(number of tested piglets × trial days).

### 2.4. Sample Collection

On the 14th day of the feeding experiment, 6 piglets each from the NC and AO3 groups were individually placed in clean crates. Rectal stimulation was performed with a sterile swab, and at least 5 g of feces were directly collected into a sterile centrifuge tube for 16S rRNA sequencing analysis.

Prior to piglet euthanasia, 10 mL of blood was obtained by venipuncture from the anterior vena cava of each fasted piglet using a heparin vacuum anticoagulant tube. The blood samples were kept at room temperature for 2–3 h before being centrifuged (3000× *g* for 15 min), and the obtained serum was stored at −20 °C.

Following this, all piglets were euthanized through the administration of pentobarbital sodium (50 mg/kg BW) via the jugular vein. Two-centimeter segments of the duodenum, jejunum, and ileum were collected and preserved in 4% paraformaldehyde for morphological observation. The chyme in the jejunum tissue was rinsed with cold saline and then frozen in liquid nitrogen for subsequent RNA and protein measurements.

### 2.5. Plasma Analysis

The plasma antioxidant and immune indexes for T-AOC, SOD, MDA, GSH, GSH-px, TNF-α, IL-1β, IL-6, and IL-12 were determined using commercial test kits (Nanjing Jiancheng Bioengineering Institute, Nanjing, China), according to the instructions from the manufacturer (absorption spectrophotometry; iMark, BIORAD, Hercules, CA, USA).

### 2.6. Mucosal Morphometry in the Jejunum

For the analysis of intestinal morphology, jejunal tissue specimens were fixed in paraformaldehyde solution at room temperature for 24 h. Subsequently, the specimens underwent dehydration using a graded series of ethanol and xylene before being processed into paraffin blocks. Cross-sections with a thickness of 5 μm were obtained from each specimen and stained with hematoxylin (HHS32, Sigma-Aldrich, St. Louis, MO, USA) and eosin (318906, Sigma-Aldrich St. Louis, MO, USA). Villus height (VH) and crypt depth (CD) were observed using a light microscope. VH represents the distance between the top of the villi and the midpoint of the connection between the villi of both crypts, while CD is the distance between the midpoint of the connection between the villous junction of both crypts and the mucosal base. The assessor, blinded to the treatment conditions, employed an optical microscope (Nikon Eclipse 80i, Nikon, Tokyo, Japan) along with NIS-Elements 3.0 imaging software to measure the values of villus height (VH) and crypt depth (CD) and the ratio of villus height to crypt depth (V:C).

### 2.7. mRNA Analysis

RNA extraction from jejunal tissue was carried out using TRIzol reagent (Invitrogen, CA, USA) following the manufacturer’s protocols. The quality and quantity of total RNA were assessed using a NanoDrop system (ThermoFisher, Waltham, MA, USA). Reverse transcription was performed using the PrimerScriptTM RT Reagent Kit (Takara, Osaka, Japan). Specific primers for real-time PCR were designed using Primer Premier 6.0 software, and their sequences are provided in Supplementary Table S1. cDNA was amplified for quantifying gene expression through real-time PCR, utilizing gene-specific primers and SYBR Green (Takara, Osaka, Japan). Gene expression changes were analyzed using the 2^−∆∆Ct^ method, with expression levels normalized to β-actin.

### 2.8. Western Blot

The frozen jejunal tissue samples were homogenized in RIPA lysis buffer containing protease inhibitors (Applygen, Beijing, China). Total protein content was determined using the BCA assay (Thermo Fisher Scientific, Rockford, IL, USA). Proteins were separated by SDS polyacrylamide gel electrophoresis and transferred to PVDF membranes (Millipore, Billerica, MA, USA). After blocking with 1 × TBST containing 5% BSA (Sigma Aldrich, St Louis, MO, USA) for 2 h at room temperature, the membranes were incubated with the corresponding primary antibodies overnight at 4 °C. The dilution ratios of p-AMPK (Cell Signaling Technology, 2535T, Boston, MA, USA), AMPK (Abcam, ab3759, Cambridge, UK), NF-κB (Proteintech, 20536-1-AP, Chicago, IL, USA), p-NF-κB (Cell Signaling Technology, 3033s, Boston, MA, USA), Claudin-1 (Abcam, ab129119, Cambridge, UK), ZO-1 (Proteintech, 21773–1-AP, Wuhan, China), and occludin (Proteintech, 27260–1-AP, Wuhan, China) were all 1:1000. The dilution ratios of JNK (Cell Signaling Technology, 9252s, Boston, MA, USA), p-JNK (Cell Signaling Technology, 4668s, Boston, MA, USA), ERK (Cell Signaling Technology, 9102s, Boston, MA, USA), and p-ERK (Cell Signaling Technology, 9101s, Boston, MA, USA) were all 1:3000. The dilution ratio of β-actin (Bioss, bs-0061R, Beijing, China) was 1:2000. After washing of membranes with 1× TBST, membranes were incubated with the HRP-conjugated goat anti-rabbit IgG (Huaxingbio Biotechnology, Beijing, China) for 1 h at room temperature. The chemifluorescene was detected with the Western Blot Luminance Reagent (Applygen, Beijing, China) using an ImageQuant LAS 4000 mini system (GE Healthcare Bio sciences AB, Sweden), and quantified by Image J software (V 1.8.0).

### 2.9. Enzyme Activity Related to Energy Metabolism and Antioxidant in Jejunum

The sample preparation processes and the jejunum enzyme related to antioxidant (T-AOC, SOD, MDA, GSH, GSH-PX) and energy metabolism (hexokinase, lactated hydrogenase, glycogen phosphorylase, hydroxyacyl-CoA-dehydrogenase, succinate dehydrogenase) activity were measured according to the manufacturer’s procedure.

### 2.10. Gut Microbiota Analysis

Microbial community genomic DNA was extracted from feces using a DNA stool mini kit (Tiangen, Beijing, China). DNA extracts were detected via agar gel electrophoresis and DNA concentration and purity were detected using a NanoDrop 2000 ultraviolet-visible spectrophotometer (Thermo Scientific, Wilmington, NC, USA). The V3–V4 region of the bacterial 16S ribosomal RNA gene was amplified with the primers 338F (5′-ACTCCTACGGGAGGCAGCA-3′) and 806R (5′-GGACTACHVGGGTWVTAAT-3′) using PCR. PCR products were isolated via 1.5% agarose gel electrophoresis and purified using the QIAquick Gel Extraction Kit (Qiagen, Dusseldorf, Germany). The purified products were sequenced using the Illumina MiSeq platform (Personal Biotechnology Co., Ltd., Shanghai, China). Qiime2 20120.11 script was employed for α- and β-diversity calculation and taxonomic community assessment [21]. α-diversity of the samples was calculated using observed species, Chao 1 index, Shannon index, and Simpson index. Principal coordinate analysis (PCoA) based on weighted UniFrac distance summarized beta diversity. Linear discriminant analysis coupling effect size (LEfSe) was applied to identify key bacterial groups among different treatments [22].

### 2.11. Statistical Analysis

In this study, Student’s *t*-test (SPSS 22.0) was employed to evaluate significant differences in growth performance between the CON and AO3 groups, with a significance threshold set at *p* < 0.05. One-way ANOVA was utilized for analyses involving more than two groups, and Tukey’s multiple comparisons were conducted, with a significance threshold set at *p* < 0.05. The data are presented as the mean ± SEM.

For the comparison of alpha diversity indices (ACE, Chao 1, Simpson, goods_coverage, and observed species) between groups, Welch’s *t*-test and the Wilcoxon rank test were performed using the R project Vegan package (version 2.5.3). Beta diversity was analyzed through principal coordinate analysis (PCoA) based on the Bray–Curtis distance. In addition, species comparison between groups was conducted using Welch’s *t*-test and the Wilcoxon rank test in the R project Vegan package (version 2.5.3).

## 3. Results

### 3.1. Growth Performance

The piglet weight results at different time points in Table 2 show that no significant difference was apparent between the different groups on days 7 and 15 of the trial. However, piglets receiving the diet supplemented with AO3 showed a trend of increasing body weight. When the experiment was carried out to day 20, the weight of the treated piglets showed a more significant difference from that of the control piglets (*p* = 0.06). It can be seen that the addition of AO3 in the diet significantly increased the body weight of piglets on days 30 and 36.

The results of average daily gain (ADG) showed that at the stages of days 1–7 and days 8–15, similar to the results for body weight, the diet supplemented with AO3 did not significantly increase ADG compared with the control diet, but there was an obvious increasing trend (*p* = 0.08). At the stage of days 31–36, the ADG of the AO3 group was significantly increased. The findings related to the feed conversion rate (FCR) indicate that during the initial week of the experiment, piglets in the treatment group that received supplementation with AO3 displayed an enhancement, while this improvement did not attain statistical significance (*p* = 0.09). Subsequently, following the second week, a notable and statistically significant increase in the FCR of piglets in the AO3 group was observed.

Furthermore, AO3 supplementation significantly decreased the diarrhea rate of piglets compared with the control group during the whole trial period, except for the period of days 21–30 and 31–36.

### 3.2. Redox Status and Inflammation in Plasma

**Figure 2:**
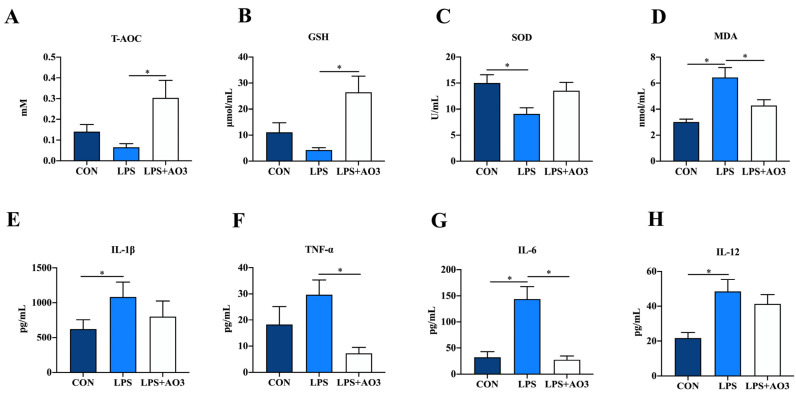
Redox status and inflammatory factors in plasma. (**A**–**D**) Relative activities of T-AOC and SOD and relative concentrations of GSH and MDA in plasma. (**E**–**H**) Content of inflammatory cytokines (TNF-α, IL-1β, IL-6, IL-12) in plasma. Control (CON): a corn–soybean meal-based diet. LPS: CON diet, treatment with LPS. LPS+AO3: CON diet + 2000 mg/kg AO3, treatment with LPS. Data are expressed as mean ± SEM. * indicates *p* < 0.05. *n* = 18 total pigs, 6 replicates per group.

The impact of dietary AO3 supplementation on the plasma redox status of piglets is shown in Figure 2A–D. Compared with the control group, SOD activity notably decreased, while MDA content significantly increased in piglets subjected to LPS induction. Compared with the LPS group, SOD activity increased and MDA content decreased in the LPS+AO3 group. However, these levels did not fully revert to those observed in the control group. As for T-AOC, AO3 displayed a distinct ability to mitigate the decline in T-AOC levels induced by LPS in piglets. Given the connection between oxidative stress and inflammation within the body, we proceeded to evaluate the expression of inflammatory factors in plasma. In the control group, plasma levels of IL-6, IL-12, and TNF-α exhibited marked increments following LPS induction. However, plasma levels of IL-1β, IL-6, IL-12, and TNF-α did not change significantly in the AO3 group after LPS induction (Figure 2E–H).

### 3.3. Redox and Inflammation in Jejunum

**Figure 3:**
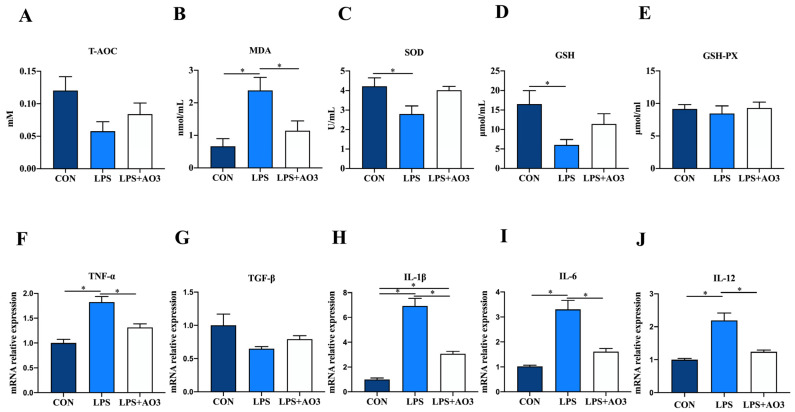
Redox status and inflammatory factors in the jejunum. (**A**–**E**) Relative activities of T-AOC, SOD, and GSH-PX and relative concentrations of GSH and MDA in jejunum. (**F**–**J**) The mRNA expression levels of inflammatory cytokines (TNF-α, TGF-β, IL-1β, IL-6, IL-12) in jejunum. Data are expressed as mean ± SEM. * indicates *p* < 0.05. *n* = 18 total pigs, 6 replicates per group.

We assessed the activity of antioxidant enzymes and the expression levels of inflammatory factor genes within the jejunum of piglets. Consistent with the serum findings, the activities of T-AOC, SOD, and GSH within the jejunum of the LPS group exhibited a significant decrease or displayed a notable tendency to decrease. Correspondingly, there was a significant increase in the MDA content (Figure 3A–E). No significant differences in antioxidant indices were observed between the AO3 group and the control group. In terms of inflammatory factors, the expression levels of *TNF-α*, *IL-1β*, *IL-6*, and *IL-12* within the jejunum of the LPS group exhibited significant upregulation, whereas TGF-β displayed no notable difference compared with the control group (Figure 3F–J). Following LPS induction, no significant difference was observed in the expression levels of inflammatory factors between the AO3 group and the control group. These findings collectively suggest that AO3 plays a crucial role in mitigating LPS-induced oxidative stress and inflammation.

### 3.4. AO3 Improved Intestinal Morphology, Barrier Integrity and Expression of Nutrient Transporters

**Figure 4:**
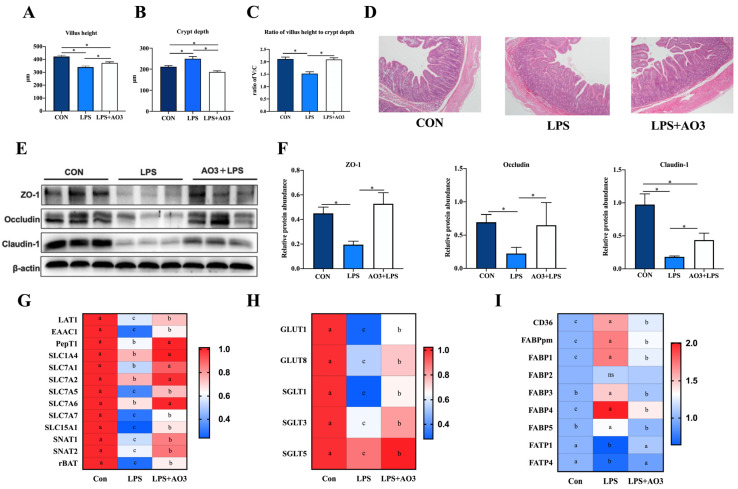
Effect of AO3 supplementation on intestinal morphology, barrier integrity, and nutrient transportation. (**A**–**D**) Villus height, crypt depth, and ratio of V:C in jejunum under different treatments. (**E**,**F**) Protein levels of intestinal barrier function in jejunum. (**G**) Relative gene expression levels of amino acid transporters. (**H**) Relative gene expression levels of amino acid transporters. (**I**) Relative gene expression levels of fatty acid transporters and fatty acid binding proteins. *n* = 18 total pigs, 6 replicates per group. Data are expressed as mean ± SEM. * indicates *p* < 0.05. Different letters indicate differences between groups.

Figure 4A–D illustrates that LPS induction resulted in a significant reduction in the villus height of the jejunum, which, notably, experienced a substantial increase following AO3 supplementation. Similarly, the crypt depth demonstrated a marked elevation upon LPS induction but exhibited a significant reduction subsequent to AO3 supplementation. LPS induced a significant reduction in the ratio of villus height to crypt depth (C:V). However, after supplementation with AO3, it was significantly increased. ZO-1, occludin, and claudin proteins are pivotal components of the intestinal mucosal barrier. Within the LPS group, significant decreases in the protein levels of ZO-1, occludin, and claudin were observed. However, following AO3 supplementation, these protein levels experienced noteworthy increases (Figure 4E,F).

Intestinal mucosal damage and disruption to the intestinal barrier frequently impede nutrient transport, and the processes are mainly regulated by specific amino acid, fatty acid, and glucose transporters. To ascertain whether LPS and AO3 influence nutrient transporters within the gut, we evaluated the expression of genes associated with glucose transporters, amino acid transporters, and fatty acid transporters. As depicted in Figure 4G–I, the gene expression of major nutrient transporters in the pig intestine was analyzed. It was revealed that the majority of glucose transporters and amino acid transporters were significantly downregulated upon LPS induction but exhibited significant upregulation after AO3 supplementation. Noteworthy genes within this category include *GLUT1*, *GLUT8*, *SGLT1*, *SGLT3*, *LAT1*, *EAAC1*, *PepT1*, *SLC7A1*, *SLC7A2*, *SLC7A5*, *SLC5A1*, *SNAT1*, *SNAT2*, and *rBAT*. Intriguingly, fatty acid transporters FATP1 and FATP4 displayed downregulation in response to LPS induction. Conversely, fatty acid binding proteins including *CD36*, *FABPpm*, *FABP1*, *FABP2*, *FABP3*, *FABP4*, and *FABP5* were significantly upregulated, with their expression substantially diminished following AO3 supplementation.

### 3.5. AO3 Alleviating Inflammation in Induced by LPS in Jejunum via TLR4/MAPK Pathway

**Figure 5:**
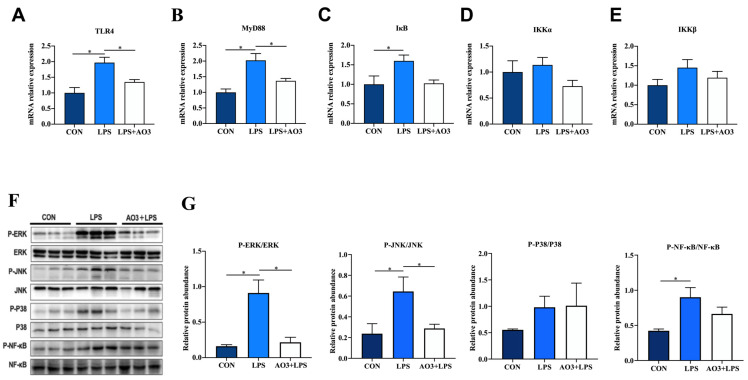
Effect of AO3 supplementation on TLR4/MAPK pathway in jejunum. (**A**–**E**) Relative gene expression levels in TLR4 signaling pathway. (**F**,**G**) Phosphorylation levels of proteins associated with MAPK signaling pathways. Data are expressed as mean ± SEM. * indicates *p* < 0.05. *n* = 18 total pigs, 6 replicates per group.

To further investigate the underlying trigger of the inflammatory response, we conducted a comprehensive analysis of genes within the TLR4 signaling pathway. Within the LPS group, substantial elevations were observed in the expression of *TLR4*, *MyD88*, *IkB*, *IKKα*, and *IKKβ*, as depicted in Figure 5A–E. In contrast, the AO3 group demonstrated notable decreases or a tendency toward reduction in the expression of these genes, relative to the LPS group. Considering that the MyD88-dependent TLR4 signaling pathway activates the MAPK pathway, we proceeded to assess protein expression within this cascade. In the LPS group, there were notable increases in the phosphorylation levels of ERK, JNK, P38, and NF-kB (Figure 5F,G). However, within the AO3+LPS group, the phosphorylation levels of MAPK-related proteins did not display significant differences compared with the control group.

### 3.6. AO3 Enhanced Jejunal Energy Metabolism through the Mitochondrial Electronic Transmission Chain (ETC) and AMPK Signaling Pathway

**Figure 6:**
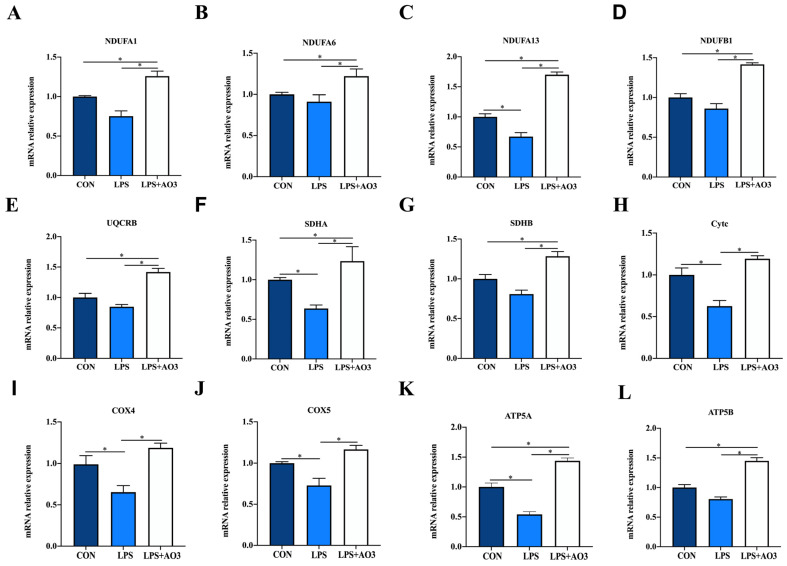
Effect of AO3 supplementation on mitochondrial electronic transmission chain (ETC). (**A**–**L**) Relative expression levels of electron transport chain (ETC)-related genes. Data are expressed as mean ± SEM. * indicates *p* < 0.05. *n* = 18 total pigs, 6 replicates per group.

**Figure 7:**
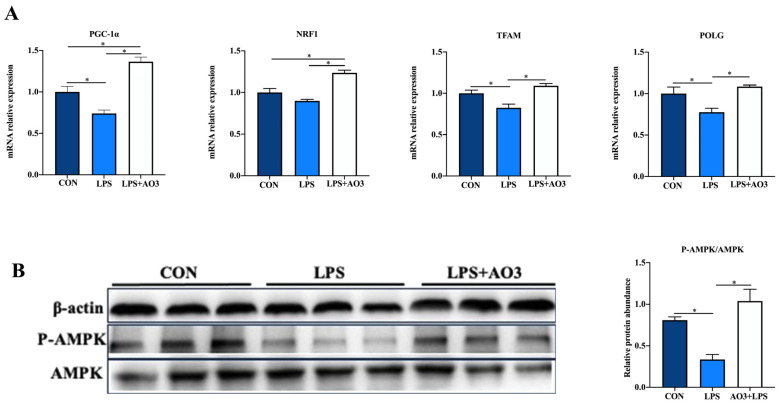
Effect of AO3 supplementation on mitochondrial electronic transmission chain (ETC) and AMPK signaling pathway. (**A**) Relative expression levels of genes associated with mitochondrial biogenesis. (**B**) Levels of AMPK phosphorylation. Data are expressed as mean ± SEM. * indicates *p* < 0.05. *n* = 18 total pigs, 6 replicates per group.

We assessed the expression of genes related to the electron transport chain (ETC) and biogenesis to elucidate the impact of LPS and AO3 on mitochondrial function. The results unveiled significant decreases in the expressions of *NDUFA1*, *NDUFA13*, *SDHA*, *Cytc*, *COX4*, *COX5*, and *ATP5A* genes subsequent to LPS injection, which were counteracted by substantial upregulation following AO3 supplementation (Figure 6). For *NDUFA6*, *NDUFB1*, *UQCRB*, and *ATP5B*, a downward trend was observed after LPS injection, but these gene expressions were significantly augmented in the AO3 group. Furthermore, LPS imposition hindered the expression of mitochondrial biogenesis-related genes such as *PGC-1α*, *TFAM*, and *POLG* (Figure 7A). In contrast, within the AO3 group, the levels of *PGC-1α* and *NRF1* were significantly upregulated in comparison to both the control and LPS groups. *TFAM* and *POLG*, although displaying a trend toward increase, did not attain statistical significance. Moreover, the ratio of phosphorylated AMPK to total AMPK (pAMPK/AMPK) was notably reduced within the LPS group. However, supplementation with AO3 facilitated the restoration of AMPK phosphorylation, thereby reversing this effect (Figure 7B). Furthermore, the impact of dietary AO3 supplementation on enzymes related to the energy metabolism of piglets are shown in Table 3. LPS challenge led to a significant reduction in the synthesis of enzymes related to energy metabolism, including hexokinase, lactated hydrogenase, glycogen phosphorylase, hydroxyacyl-CoA-dehydrogenase, and succinate dehydrogenase, which was subsequently restored by AO3 supplementation.

### 3.7. AO3 Improved Gut Microbial Diversity and Composition in Piglets

**Figure 8:**
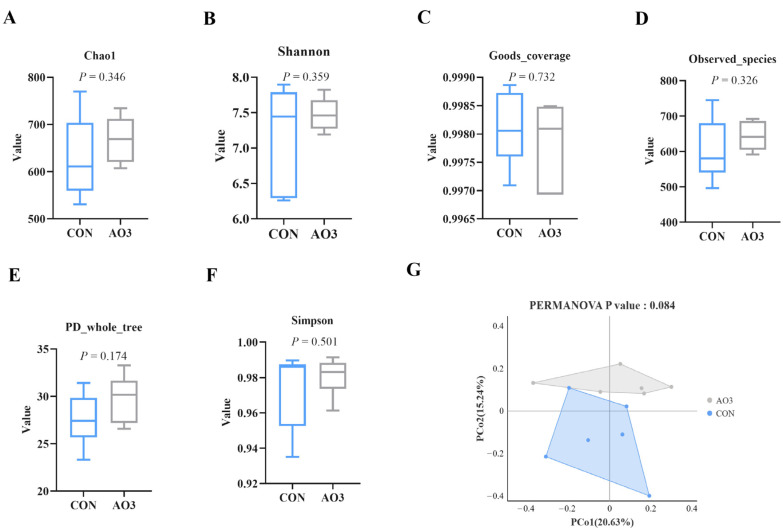
Effects of AO3 supplementation on the fecal microbial diversity of piglets. (**A**–**F**) Comparison of the Chao1 index, Shannon index, good coverage, observed species, Simpson index, and PD whole tree metrics between the CON group and AO3 group. (**G**) Beta diversity of the fecal microbiota between the control group and AO3 group. CON: a corn–soybean meal-based diet. AO3: CON diet+ 2000 mg/kg AO3. *n* = 12 total pigs, 6 replicates per group.

**Figure 9:**
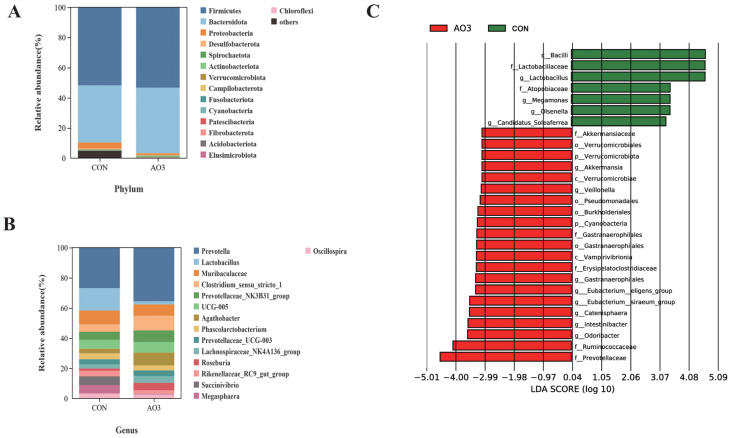
Effects of AO3 supplementation on piglets’ fecal microbial composition. The relative abundances at different phylum (**A**) and family (**B**) levels in the fecal microbiota of CON and AO3 groups. (**C**) Linear discriminant analysis coupled with effect size (Lefse) of fecal microbiota compositions in CON and AO3 groups. *n* = 12 total pigs, 6 replicates per group.

The microbiota diversity and compositions of feces of piglets at day 14 were determined by deep sequencing the 16S rRNA genes. To assess fecal microbial community structure, we calculated the alpha and beta diversity values (Figure 8). Notably, the alpha diversity index of the AO3 group, as measured by the Chao1 index, Shannon index, observed species, and PD whole tree metrics, demonstrated increased values when compared with the control group. These differences did not reach statistical significance. Moreover, outcomes from the principal coordinates analysis employing Bray–Curtis distances indicated discernible differentiation of the AO3 group (*p* = 0.084) at day 14 in contrast to the control group. A detailed analysis at the phylum level unveiled the consistent predominance of Firmicutes (53.41% vs. 51.87%) and Bacteroidetes (43.43% vs. 37.77%) within the gut microbiota composition of the piglets. Minor phyla included Proteobacteria (1.12% vs. 3.97%) and others (0.33% vs. 4.90%) (Figure 9A). At the family level, dominant bacterial families encompassed Prevotellaceae (33.26% vs. 25.10%), Lachnospiraceae (19.72% vs. 10.72%), Oscillospiraceae (8.54% vs. 9.49%), and Ruminococcaceae (6.89% vs. 4.11%) (Figure 9B). Furthermore, notable distinctions in microbial communities emerged across the various treatment groups (Figure 9C). Noteworthy bacterial taxa such as f_Prevotellaceae, f_Ruminococcaceae, f_Marinifilaceae, g_Odoribacter, f_Peptostreptococcaceae, g_Intestinibacter, f_Erysipelotrichaceae, and g_Catenisphaera exhibited greater abundance within the AO3 treatment group compared with the control group.

## 4. Discussion

Due to their inherent natural antibacterial properties and their potential to stimulate growth, organic acids and essential oils have garnered considerable attention in the realm of animal nutrition in recent years [23,24,25]. It is well established that benzoic acid and essential oils, whether used independently or in combination, offer substantial benefits to young animals, including weaned pigs, chickens, and rabbits [26,27]. This is attributed to their antibacterial and anti-diarrheal properties, effectively inhibiting the proliferation of harmful intestinal bacteria. Here, we conducted a detailed evaluation of the effect of a complex comprising benzoic acids, fumaric acids, and essential oils on weaned piglets, using weekly body weight measurements. The addition of AO3 greatly improved average daily gain (ADG) and feed conversion ratio (FCR) throughout the entire trial period, with noticeable enhancements in growth performance observed every week starting from day 7. These results were consistent with the findings related to the complex of benzoic acids and essential oils but provide additional information that significant effects can be achieved with as little as one week of supplementation [18]. It is interesting to note that a noticeable improvement in average daily feed intake (ADFI) was first observed in the second week, possibly indicating that this benefit requires some time, suggesting that supplementation needs at least one week to enhance feed intake. In line with previous observations [28,29], we also noticed a significant reduction in the diarrhea rate during both the initial 15 days and the subsequent period of days 16–36. The lack of difference during days 21–30 could be attributed to the use of medication to control porcine viral diarrhea (PVD) on the farm, which may have also influenced the occurrence of diarrhea. In summary, our study confirmed for the first time the beneficial effects of essential oils, fumaric acids, and benzoic acids on growth performance and the mitigation of weaning-related stress-induced diarrhea in piglets.

Lipopolysaccharide (LPS) has the ability to trigger inflammatory responses and disturb the delicate balance within the gastrointestinal system, making it a useful tool for simulating weaning stress [30]. Weaning stress, characterized as an acute stressor, shares similarities with the physiological reactions induced by the LPS challenge model, including diarrhea, reduced feed intake, and inflammatory responses [31]. Therefore, incorporating the LPS model into our study aimed to explore how AO3 enhances productivity by bolstering stress resilience. We found that the AO3 complex significantly ameliorated LPS-induced intestinal morphological damage, as evidenced by increased villus height and decreased crypt depth, indicating its potential in maintaining intestinal morphology. Toxins and metabolites produced by pathogenic bacteria can compromise the integrity of the intestinal mucosal barrier, leading to impaired nutrient absorption and piglet diarrhea [32]. ZO-1, occludin, and claudin are integral proteins within the intestinal barrier, playing crucial roles in maintaining barrier integrity, regulating selective permeability, and stabilizing tight junctions [33]. In the current study, the LPS challenge led to the downregulation of all three key proteins, while AO3 supplementation notably reversed this reduction, underscoring their protective roles in maintaining intestinal barrier integrity. These results are in line with previous findings, where the dietary supplementation of a 3000 mg/kg blend of organic acid was shown to increase the relative expression of genes such as *claudin-1*, *occludin-2*, and *mucin-2* in the jejunum of piglets [34]. The reduced diarrhea rate among piglets in the AO3 group may be attributed to the concurrent enhancement of the intestinal mucosal barrier.

The maintenance of redox balance is crucial for preserving cellular and tissue integrity, protecting against oxidative damage, and contributing to overall health [35]. Weaning stress is known to frequently induce a redox imbalance in piglets, leading to organ damage and increased vulnerability to various diseases [36]. Our study observed that AO3 significantly improved or exhibited a tendency to improve the REDOX state of piglets subjected to LPS in both serum and intestinal samples of piglets. In line with the enhanced antioxidant capacity, AO3 supplementation notably attenuated LPS-induced inflammation, characterized by a significant reduction in elevated cytokines TNF-α, IL-1β, IL-6, and IL-12, bringing their levels closer to those of the control group in both serum and intestine. These results are consistent with prior research, which demonstrated that supplementation with organic acid mixture alleviated redox disturbances and inflammation induced by E. coli challenge in piglets [37]. We further examined the activation of the MAPK signaling pathway, which mediates inflammation by triggering the expression of proinflammatory genes in response to external stimuli, to explore the mechanism by which AO3 alleviates LPS-induced inflammation [38]. It is worth noting that all three major branches of the MAPK signaling pathway, including ERK, JNK, and p38, exhibited upregulated activation in response to LPS challenge, which AO3 administration successfully restored [39]. NF-κB is another crucial signaling pathway participating in regulating inflammation, by translocating into the nucleus and initiating the transcription of proinflammatory genes in response to various stimuli, and its activation induced by LPS was also suppressed in the current study. The gene expression of other key players, such as *TLR4*, *MyD88M*, and *IκB*, involved in the regulation of MAPK and NF-κB signaling was also found to be altered among the three groups. This further emphasizes the involvement of these two signaling pathways in the alleviation of the LPS-induced inflammatory response by AO3.

Nutrient absorption in the intestine mainly relies on energy-intensive active transport, where specialized proteins actively transport nutrients against concentration gradients, necessitating cellular energy in the form of ATP [40]. Mitochondria serve as essential hubs for cellular metabolism and energy production, with the operation of electron transport chain complexes (CI, CII, CIII, CIV, CV) playing a central role in mitochondrial energy synthesis [41]. Mitochondrial dysfunction has been identified as a contributor to the development of intestinal disorders, disrupting normal cellular processes and gut function [42,43]. In this study, LPS challenge led to a significant reduction in the expression of genes involved in electron transport chain complexes, which was subsequently restored by AO3 supplementation. Adenosine monophosphate-activated protein kinase (AMPK), a crucial regulator of cellular energy metabolism, contributes to maintaining intracellular ATP balance [44]. Similar trends were also observed in the activation of this signaling pathway, which limits energy-intensive anabolic processes while stimulating productive catabolic processes to maintain energy balance. Additionally, the activities of enzymes responsible for catalyzing energy metabolism [45], including hexokinase, lactated hydrogenase, glycogen phosphorylase, hydroxyacyl-CoA-dehydrogenase, and succinate dehydrogenase, exhibited comparable patterns. This observation suggests that AO3 may have a potential role in mitigating insufficient energy production resulting from mitochondrial metabolic disturbances induced by LPS. The active transport of nutrients such as amino acids, glucose, and fatty acids within the intestine necessitates ATP support. The marked restoration in nutrient transporter expression within the AO3 group might stem from the repaired mitochondrial function and enhanced energy production. Notably, genes associated with fatty acid binding proteins (FABPs) were upregulated in the LPS group. FABPs, belonging to the intracellular lipid binding protein family, significantly contribute to the physiological regulation of the uptake, transport, and metabolism of long-chain fatty acids in animals [46,47]. We hypothesize that the upregulation of FABPs-related genes in the LPS group might be due to the disorder of fat metabolism produced by intestinal cells under stress.

The gut microbiota play a profound role in influencing host health by safeguarding the integrity of the intestinal barrier, and their metabolites can translocate from the gut to remotely impact the function of other organs [48,49,50]. The results of our current study indicate that the addition of AO3 effectively preserves intestinal integrity and function against LPS challenge, raising our curiosity about whether this benefit is associated with gut microbiota balance. The current findings align with prior research, such as Ma’s study, where it was demonstrated that a dietary blend of organic acid supplementation increased the Sobs, Ace, and Chao indices in cecum and colon microbiota [51]. β-diversity analysis is pivotal for comparing species diversity among distinct microbial communities, unveiling similarities or disparities in community composition across various sample groups [52]. Here, we found that AO3 exerted an impact on the fecal microbial composition of piglets according to β-diversity analysis (*p* = 0.084). We did not observe a significant difference in α diversity between the AO3 group and the control group, possibly due to the relatively short 14-day feeding period. Notably, we found that dietary AO3 supplementation heightened the abundance of f_Prevotellaceae within piglet feces. Prevotellaceae-driven intestinal microbiota has been associated with various animal traits, including feed intake, feed efficiency, weight gain, and diarrhea incidence [53,54,55]. These associations suggest that *Prevotella* plays a pivotal role in improving growth performance and mitigating intestinal stress and injury caused by LPS challenge upon AO3 supplementation. Collectively, the enhancement of intestinal microbial structure, particularly the specific enrichment of *f_Prevotellaceae*, is one of the key mechanisms through which AO3 improves piglet intestinal function during weaning.

## 5. Conclusions

In conclusion, dietary AO3 supplementation positively affects growth performance of weaning piglets though modulating microbiota composition and enhancing intestinal barrier integrity, nutrient uptake, and energy metabolism, as well as alleviating oxidative stress and inflammation upon LPS stimulation. This study represents the first evaluation of a complex comprising formic acid, benzoic acid, and essential oils, offering a solid foundation and inspiration for the development of beneficial functional additives aimed at improving gut health in both human and animal applications.

## Figures and Tables

**Table 1:** Composition and nutrient profile of the basal diets (as fed basis).

Ingredients	Content(%)	Nutritional level	Content(%)
Corn	47.25	Calculated values	
Wheat standard powder	7.50	Dry matter	15.05
Low protein whey powder	7.50	Crude protein	19.20
Cane sugar	2.50	Crude fat	8.03
Glucose	1.25	Crude fiber	1.76
Peeled soybean meal	8.50	Crude ash	3.01
Extruded soybean	10.00	calcium	0.77
Peruvian fishmeal	7.00	Total phosphorus	0.53
Soybean oil	2.00	Available phosphorus	0.34
Yeast extract	2.50	Lysine	1.35
Choline chloride (50%)	0.10	Methionine + cystine	0.74
Calcium hydrogen phosphate	0.38	Threonine	0.79
Stone powder	0.80	Tryptophan	0.22
Salt	0.30		
L-lysine hydrochloride (98.5%)	0.50		
D-methionine (99%)	0.22		
L-threonine (98.5%)	0.25		
L-tryptophan (98.5%)	0.02		
L-valine (98.5%)	0.10		
^1^ Vitamin premix	0.035		
^2^ Mineral premix	0.23		
Chaff mixing	1.065		
Total	100.00		

^1^ Provided vitamins per kg of diet: vitamin A 11375 IU; vitamin D3 3500 IU; vitamin E 26.3 IU, vitamin K3 3.5 mg; vitamin B1 3.5 mg; riboflavin 8.8 mg; vitamin B6 5.4 mg; vitamin B12 0.03 mg; pantothenic acid 17.5 mg; niacin 35 mg; folic acid 1.75 mg; biotin 0.14 mg. ^2^ Provided trace mineral elements per kg of diet: copper (copper glycinate) 64.4 mg; iron (iron glycinate) 165 mg; manganese (manganese glycinate) 48 mg; zinc (zinc glycinate) 48 mg; selenium (yeast selenium) 0.54 mg; iodine (calcium iodate) 0.68 mg; cobalt (cobalt sulfate) 0.1 mg.

**Table 2:** Weight, average daily gain, average daily feed intake, feed conversion rate, and diarrhea rate of piglets at different weighing stages.

Items	Con	AO3	SEM	*p*-Value
Body weight, kg				
d 1	6.61	6.57	0.21	0.98
d 7	9.76	10.02	0.31	0.08
d 15	12.63	13.25	0.44	0.09
d 20	15.56	16.27	0.52	0.06
d 30	21.03 ^a^	22.99 ^b^	0.61	0.04
d 36	25.18 ^a^	27.84 ^b^	0.64	0.05
Days 1 to 7				
ADG, g	455	490	15.18	0.08
ADFI, g	532	529	15.87	0.937
FCR, g/g	1.17	1.08	0.007	0.09
Diarrhea rate, %	17.34 ^a^	13.14 ^b^	0.76	0.04
Days 8 to 15				
ADG, g	410	464	16.47	0.08
ADFI, g	652	687	22.38	0.07
FCR, g/g	1.59	1.48	0.02	0.05
Diarrhea rate, %	20.39 ^a^	17.23 ^b^	0.51	0.03
Days 16 to 20				
ADG, g	586	604	24.42	0.156
ADFI, g	890	863	32.35	0.236
FCR, g/g	1.52	1.43	0.05	0.132
Diarrhea rate, %	8.52 ^a^	5.2 ^b^	0.46	0.041
Days 21 to 30				
ADG, g	547 ^a^	672 ^b^	21.18	0.044
ADFI, g	886 ^a^	994 ^b^	36.76	0.048
FCR, g/g	1.62 ^a^	1.48 ^b^	0.03	0.045
Diarrhea rate, %	7.45	5.46	0.35	0.411
Days 31 to 36				
ADG, g	691 ^a^	808 ^b^	10.92	0.029
ADFI, g	1182	1236	26.58	0.098
FCR, g/g	1.71 ^a^	1.53 ^b^	0.03	0.031
Diarrhea rate, %	4.75	3.41	0.43	0.028
Days 1 to 36				
ADG, g	516 ^a^	582 ^b^	14.98	0.041
ADFI, g	770	810	19.45	0.082
FCR, g/g	1.49 ^a^	1.39 ^b^	0.03	0.043
Diarrhea rate, %	12.64 ^a^	7.80 ^b^	0.6	0.039

Note: ADG = average daily gain; ADFI = average daily feed intake; FCR = feed conversion rate. Values with different lowercase superscripts differ (*p* < 0.05).

**Table 3:** Enzyme activity related to energy metabolism in jejunum.

Enzyme Activity (U/g Protein)	Con	LPS	LPS+AO3	*p* Value
Hexokinase	168.6 ± 6.1 ^a^	127.7 ± 10.1 ^c^	153.8 ± 9.2 ^b^	<0.001
Lactated hydrogenase	989 ± 26.3 ^a^	793.3 ± 83.9 ^b^	878.3 ± 68.1 ^a^	<0.001
Glycogen phosphorylase	454.5 ± 21.6 ^a^	265.2 ± 19.2 ^b^	407.3 ± 29.2 ^a^	<0.001
Hydroxyacyl-CoA-dehydrogenase	205.3 ± 29.4 ^a^	89.4 ± 12.1 ^b^	148.2 ± 12.8 ^c^	<0.001
Succinate dehydrogenase	798.2 ± 32.7 ^a^	587.3 ± 57.2 ^b^	698.3 ± 60.2 ^c^	<0.001

Note: Data are expressed as mean ± SEM. Values with different lowercase superscripts differ (*p* < 0.05).

## Data Availability

The data that support the findings of this study are available on requestfrom the corresponding author.

## Supplementary Materials

The following supporting information can be downloaded at: https://www.mdpi.com/article/10.3390/antiox13020246/s1, Table S1: Primers uesd in this study.
