# Supplementary material for: A Mixture of Formic Acid, Benzoic Acid, and Essential Oils Enhanced Growth Performance via Modulating Nutrient Uptake, Mitochondrion Metabolism, and Immunomodulation in Weaned Piglets
Source: Antioxidants (Basel). 2024 Feb 19;13(2):246. doi: 10.3390/antiox13020246 (PMC10886008; doi:10.3390/antiox13020246)
Supplement: Supplementary file 1 [file antioxidants-13-00246-s001.zip › antioxidants-2781903-supplementary.pdf]

Table S1 Primer sequences used in real-time PCR

Gene Name	Primer Sequence(5’-3’)	
GLUT1	GATGAAGGAGGAGTGCCG	CAGCACCACGGCGATGAGGAT
GLUT8	TATGTTGCGGATGCTATGGG	ATGAGATGGTCCCAATTTCG
SGLT1	ATTCTTTGGTGGGATGCTTG	CATCATCGTCCTGGTCGTC
SGLT3	CCTTGGGATTGGACCTTTATC	CGTTTGCGGAGGGTGTCTGTGT
SGLT5	GCGTCCAGATCGGCAGAACC	GCGTCCAGATCGAGAACCT
LAT1	GCCCATTGTCACCATCATC	GAGCCCACAAAGAAAAGC
SLC1A4	AGACCTCTCTTTGATCCTGC	TGTTTCCTCCTCTGATTTGCA
SLC7A1	GCCTGAGAGCAAGACCAAA	GCCGTAGCCGAAGTAGATGA
SLC7A2	GCCCCAGAATCAGCAAAAA	GATGCTGAAGGCTGGCAAAA
SLC7A5	CTCTTCCTGATCGCCGTCTC	CTTCTGACACAGGACGGTCGT
SLC15A1	TTCGGTTATCCCTTGAGCAT	GTGGTAGATGGCAGTGGACAGG
SNAT1	AAGAACCTGGGCTATCTCG	TGTTGCGTTAGGACTCGTTG
SNAT2	GTTACCTTTGGTGATCCAGG	ACCAATGACACCAGCAGAACC
rBAT	TTTCCGCAATCCTGATGTTC	GGGTCTTATTCACTTGGGTC
CD36	GGACTCATTGCTGGTGCTGT	GTCTGTAAACTTCCGTGCCTGT
FABP1	CAGAAGGGGAAGGATGTCA	CTCCCCAGTCATGAACTCCA
FABP2	ACATCACTTGTGCGGGGAAA	CCAGTGTCACGATGGACTTG
FABP3	CTGGGAGTGGAGTTTGATG	CCATGGGTGAGTGTCAGGAT
FABP4	TGAAAGGTGTCACGGCTAC	TCGGGACAATACATCCAACAGG
FABP5	CTGGGACAGAAGTTTGAAG	GACCCGAGTGCAGGTGACATT
FATP1	GGCAACAGACGTGATCTATG	AGCGGCTGGCTGAAAACT
FATP4	AGCCGCATCCTGTCCTTT	GACATCCTTGGCGATCTTTT
TNF-α	GCCCTTCCACCAACGTTTTC	CAAGGGCTCTTGATGGCAGA
TGF-β	GGCACTGCTTCCCGAATGTC	ACCGCAACAACGCCATCTATGAG
IL-1β	ATTCAGGGACCCTACCCTCT	ATCACTTCCTTGGCGGGTTC
IL-6	ACAAAGCCACCACCCCTAA	CGTGGACGGCATCAATCTCA
IL-12	CAACCCTGTGCCTTAGCAGT	AGAGCCTGCATCAGCTCAGT
TLR4	GAGCCGGAAGGTTATTGTGG	GAGCCGGAAGGTTATTGTGGTATG
My-D88	AGCAGAACCAGGAGTCCGA	GGGCAGTAGCAGATAAAGGCATC

*I*κB	AGACCCAGGAGTGTTCACA	GTCACCAGGCGAGTTATAGCT
*IKK*α	GGATATGAGGAAGCGGCATG	CCTGATACTGGCACTTCGGACAA
*IKK*β	GTCACCAGGCGAGTTATAGC	CTTCACCTGTCAAACCTGGCT
PGC-1α	GCTTGACGAGCGTCATTCAG	GGTCTTCACCAACCAGAGCA
NRF1	GAAGCTGTCCAGGGGCTTTA	ATCCATGCTCTGCTACTGGG
TFAM	AGCGAGGTCTGAAGAGTTGC	TTGCACCCGTAGACAAAGCA
POLG	CTGTCAGATGAGGGCGAGTG	ACTTCTTCCGTCGTGACTTTCT
NDUFA1	GCTTCCGGGGAAGGAATCAA	CCGGGGAGAATTTCGAACCA
NDUFA6	TCTCAGAGCCTTGCATGTCG	AAGCCATCCAGCATCGTACC
NDUFA13	ATGAAGGATGTGCCGGACTG	CCATAGGTGGCGCTGAGAAT
NDUFB1	TGCCTTCCGGAACAAGAGT	GCAATTCAGCCACAGCCTTT
SDHA	CAATAAGAGGTCGTCGGCC	AGAGAGACCAAACGCAGCTC
SDHB	TCCTATGGTGTTGGATGCGT	AGTGTTGCCTCCGTTGATGT
UQCRB	CATCAGGCAACGCTTCTGTC	TATACCCTCCAGCCACTTGC
Cytc	CTGGGGAGAGGAGACACTG	AGGCGGTGGCCAACTTTTAC
COX4	CCAAGTGGGACTACGACAA	CCTGCTCGTTTATTAGCACTGG
COX5	ATCTGGAGGTGGTGTTCCTA	GTTGGTGATGGAGGGGACTAAA
ATP5A	ACGCCATTGATGGAAAGGG	TGGTTCCCGCACAGAGATTC
ATP5B	CATGTTGGGCTTTGTGGGTC	ATAGTCTCTGGCAGGCTGGA
β-Actin	TGCGGGACATCAAGGAGAA	AGTTGAAGGTGGTCTCGTG
